# Potential process ‘hurdles’ in the use of macroalgae as feedstock for biofuel production in the British Isles

**DOI:** 10.1002/jctb.5003

**Published:** 2016-05-10

**Authors:** John J Milledge, Patricia J Harvey

**Affiliations:** ^1^ Algae Biotechnology Research Group, School of ScienceUniversity of GreenwichCentral Avenue, Chatham MaritimeKentME4 4TBUK

**Keywords:** seaweed, algae, macroalgae, biorefining, biofuel, bioenergy, gasification

## Abstract

This review examines the potential technical and energy balance hurdles in the production of seaweed biofuel, and in particular for the MacroBioCrude processing pipeline for the sustainable manufacture of liquid hydrocarbon fuels from seaweed in the UK.

The production of biofuel from seaweed is economically, energetically and technically challenging at scale. Any successful process appears to require both a method of preserving the seaweed for continuous feedstock availability and a method exploiting the entire biomass. Ensiling and gasification offer a potential solution to these two requirements. However there is need for more data particularly at a commercial scale. © 2016 The Authors. *Journal of Chemical Technology & Biotechnology* published by John Wiley & Sons Ltd on behalf of Society of Chemical Industry.

**Abbreviations**
AD: Anaerobic digestion
dw: Dry weight
EROI: Energy return on energy investment
ESPRC: Engineering and Physical Sciences Research Council
IMTA: Integrated multi‐trophic aquaculture
HHV: Higher heating value
LCA: Life cycle assessment
MBC: MacroBioCrude
SCWG: Supercritical water gasification
TS: Total solids
VS: Volatile solids
wt: Weight

## INTRODUCTION

Algae are a diverse range of aquatic ‘plants’, comprising both unicellular and multi‐cellular forms, which generally possess chlorophyll, but are without true stems and roots. The algae can be divided by size into two groups: macroalgae commonly known as ‘seaweed’ and microalgae, microscopic single cell organisms ranging in size from a few micrometres to a few hundred micrometres (µm).^1^

Macroalgae or seaweeds have been used by mankind for generations as a food and for soil conditioning or fertiliser. Global utilisation of macroalgae is a multi‐billion dollar industry^2^ with world production of seaweed increasing, between 1970 and 2010 from < 2 million to 19 million tonnes fresh weight.^3^ Despite, the focus of much recent research being on microalgae rather than macroalgae, the macroalgal non‐fuel industry is currently 100 times bigger globally in wet tonnage terms than the microalgal industry.^4^, ^5^, ^6^ The current uses of seaweeds include human foods, fertilisers, phycocolloids and cosmetic ingredients,^7^ with Asia being the main market.^8^, ^9^ However, seaweed is still considered an underutilised resource worldwide.^10^

Algae, unlike terrestrial crops, do not require agricultural land for cultivation and many species grow in brackish or salt water avoiding competition for land and fresh water required for food production.^4^, ^11^ The potential biomass yield of algae per unit area is also often higher than that of terrestrial plants with, for example, brown seaweeds grown ‘under cultured conditions’ having yields of ∼13.1 kg dry weight (dw) m^−2^ yr^−1^compared with ∼10 kg dw m^−2^ yr^−1^ from sugarcane.^12^, ^13^ This high potential biomass yield and growth systems that do not compete for land or freshwater with agricultural crops has led to research interest in the use of macroalgae as a source of biofuel.^14^, ^15^ Nevertheless, despite their obvious potential, there are no economically‐viable commercial‐scale quantities of fuel from macroalgae.

**Figure 1:**
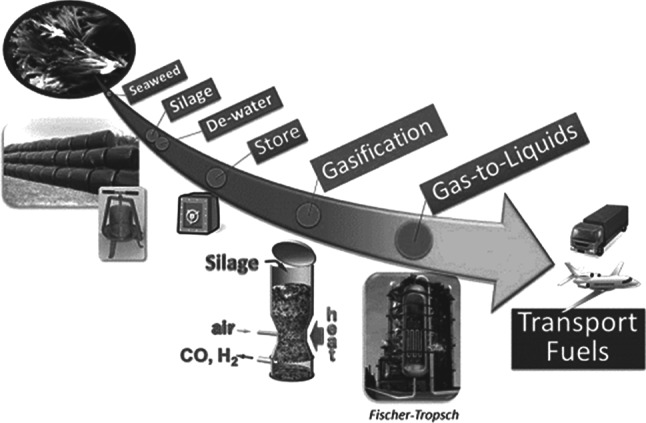
MacroBioCrude process overview (courtesy of Philip W. Dyer, University of Durham).

The MacroBioCrude (MBC) funded by a £2.3 million grant from the Engineering and Physical Sciences Research Council (EPSRC) is a cross‐discipline project to establish an integrated supply and processing pipeline for the sustainable manufacture of liquid hydrocarbon fuels from seaweed (or macroalgae). It is examining methods to overcome the seasonal supply and the high water content of seaweed, and modification of existing fossil fuel technologies of gasification and Fischer–Tropsch to use seaweed as feedstock to produce drop‐in transport fuels. An overview of the process being examined by MacroBioCrude consortium is shown in Fig. 1. The object of this review is to examine the potential technical and energy balance hurdles in the production of seaweed in the UK and in particular via the MBC proposed process.

The process operations used for algal‐derived fuel production can be grouped into four main areas:

- cultivation (including seedling production);
- harvesting;
- post‐harvest treatments including cleaning, size reduction, preservation and storage;
- energy extraction.

Any future successes of macroalgal‐derived fuel will be dependent on achieving an optimised, energy efficient process in each of these four areas. In particular, the establishment of efficient methods for the deployment of the lines from which seaweed is grown artificially and subsequent harvesting of the biomass are essential as these have found to be the main energy‐consuming operations in a life cycle assessment (LCA) study of macroalgae‐to‐fuels production processes.^16^

## CULTIVATION

It is generally recognised that the cultivation of seaweed is the only way in which supply can be matched to current and future demand around the world.^17^ Modern seaweed cultivation began in the early 1950s when the ‘summer sporeling method’ for the production of Laminaria juveniles was developed for growing‐on in raft cultivation in China.^18^ Although, seaweed cultivation has a history of only a few decades, it has developed rapidly^17^ with macroalgal production in 2006 from aquaculture accounting for 15.1 million wet tonnes of the annual world production, compared with the harvest from wild stock totalling about 1.1 million wet tonnes,^8^, ^19^ and reaching 20 million wet tonnes of farmed seaweed in 2010.^20^ However, compared with the global cultivation areas of land crops and areas of shallow sea potentially available, worldwide seaweed cultivation is relatively small, only 200 000 ha in 1999.^17^

Today China accounts for >70% of the world's total macroalgal production.^8^, ^19^ In contrast, cultivation of macroalgae is currently in its infancy in Europe with commercial exploitation of cultivated seaweed being found in France and Spain and on an experimental basis in Ireland and Norway.^21^ In the UK the exploitation of macroalgae has been mainly limited to the exploitation of wild seaweed for higher value gourmet ingredients,^22^ although seaweed is now also beginning to be commercially farmed at limited scale in the UK for food use at Loch Fyne, producing in 2013, three tonnes dw of Alaria esculata grown on 2 km of lines in a site area of 5 ha.^23^ Plans were unveiled by the Scottish Association for Marine Science (SAMS), in August 2015, for a commercial‐scale 1 ha demonstration seaweed farm off the Argyll coast in Scotland. The facility will grow seven native species of seaweed with a total combine yield of 24 tonnes year^−1^.^24^

The cultivation of macroalgae can be divided into two stages:

- production of juvenile algae;
- growing‐on the juveniles to produce biomass.

Xiu‐geng et al. 17 have suggested that improved techniques are required for both juvenile cultivation and growing‐on adult plant to further exploit the potential of microalgae and reduce labour costs.

### Species selection

The three main algal phyla are Rhodophyta (red algae), Phaeophyta (brown algae) and Chlorophyta (green algae).^25^ Worldwide 221 species of macroalgae are currently known to be exploited by humankind with 66% of the species used as food.^26^ However, the majority of algal biomass comes from a relatively small number of species^21^ with five genera, Laminaria (reclassified as Saccharina for some species), Undaria, Porphyra, Euchema, and Gracilaria, representing 76% of the total tonnage for cultured macroalgae.^19^ Laminaria, Undaria, Porphyra, Gracilaria, Eucheuma and Kappaphycus species all have annual production of more than one million wet tonnes for non‐fuel use.^17^ The main species cultivated in Europe are S. latissima and U. pinnatifida.^27^

A number of species of seaweed are currently exploited on a relatively small scale in the British Isle compared with that in Asia. Table 1 gives a list of the seaweed species exploited in Ireland and estimates of their annual harvest.

**Table 1:** Species commercially harvested in Ireland and estimated annual seaweed harvest^28^

Species	Annual harvest (tonnes)
Ascophyllum nodosum	25 000
Fucus serratus	200
Palmaria palmata	<100
Chondrus crispus / Mastocarpus stellatus	<100
Laminaria digitata	<150
Himanthalia elongata, Saccharina latissima, L. hyperborea, Ulva sp., Porphyra sp., F. vesiculosus, Alaria esculenta.	<10

However, species currently exploited for non‐fuel uses may not be ideal for growth for conversion to biofuel. A seaweed feedstock for biofuel production should have the following characteristics:

- readily propagate vegetatively or have a simple reproductive cycle that allows seedling production;
- readily attach to substrate;
- have a rapid growth rate;
- resistant to attack by bacteria, fungi, epiphytes and grazers;
- resistant to damage and removal by tide, currents and storms;
- easily harvested;
- have a high heating or calorific value;
- have low moisture content;
- low ash, sulphur and nitrogen content.

Ascophyllum nodosum is the species with the current largest annual harvest in the British Isles.^28^, ^29^ The ultimate composition and higher heating value (HHV) of Ascophyllum nodosum is shown in Table 2. It has a low ash content and favourable ultimate composition and HHV compared with other seaweeds that have been considered as feedstock for biofuels shown in Table 3. However, Ascophyllum nodosum and some other intertidal seaweed species have higher polyphenolic content (up to 14%), which can inhibit bacteria that may be involved in the preservation and conversion of seaweeds to fuel, compared with other sub‐tidal species of seaweed (sub‐tidal kelps <2%).^8^, ^30^ Ascophyllum nodosum, therefore, may not be suitable for cultivation to fuel.

**Table 2:** Compositional and higher heating value (HHV) data for Ascophyllum nodosum

	Ash	Carbon	Hydrogen	Oxygen	Nitrogen	Sulphur	HHV
	% dw	% dw	% dw	% dw	% dw	% dw	MJ kg^−1^ dw
A. nodosum	21.1	37.3	5.2	31.0	3.0	2.5	15.6

**Table 3:** Compositional and higher heating value (HHV) data for some species of seaweed being considered as potential biofuels

	Ash	Carbon	Hydrogen	Oxygen	Nitrogen	Sulphur	HHV
	% dw	% dw	% dw	% dw	% dw	% dw	MJ kg^−1^ dw
Fucus vesiculosus 6	22.82	32.88	4.77	35.63	2.53	2.44	15.0
Chorda filum 6	11.61	39.14	4.69	37.23	1.42	1.62	15.6
Laminaria digitata 6	25.75	31.59	4.85	34.16	0.9	2.44	17.6
Fucus serratus 6	23.36	33.5	4.78	34.44	2.39	1.31	16.7
Laminaria hyperborea 6	17.97	34.97	5.31	35.09	1.12	2.06	16.5
Macrocyctis pyrifera 6	38.35	27.3	4.08	34.8	2.03	1.89	16.0
Enteromorpha prolifera 31	30.1	28.75	5.22	32.28	3.65	0	12.2*
Laminaria saccharina 32	24.2	31.3	3.7	36.3	2.4	0.7	11.1*

* Calculated using a version of the DuLong equation^33, 34^

A previous extensive review for the Crown Estates concluded that, although a wide range of seaweeds can be grown in the UK, L. saccharina, L. hyperborea (as well as other laminariacea), S. polyschides and Alaria species are promising candidates for biofuel production in the British Isles, due to their fast growth rates and yields in cultivation.^8^ Trial cultivation of Laminaria hyperborea, L.digitata, S. polyschides and S. latissimi have all been attempted in Ireland or Scotland.^15^ The recent EnAlgae project, examining the cultivation of seaweed for biofuels, has focused much of its research on the growth S. latissima in both France and Ireland.^27^

### Production of juvenile algae

Juvenile macroalgal cultivation can be divided into two categories:^17^, ^27^

- sexual propagation starting from microscopic haploid spores;
- vegetative propagation starting from macroscopic diploid algal fragments.

In the production of juvenile algae a number of factors need to be controlled, which include: elimination of disease and predators by sterilisation of incoming seawater; temperature; salinity; water movement; and lighting, with blue light found to be particularly important in the reproductive cycle of seaweed,^28^ and a minimum light requirement of 1 µmol m^−2^ s^−1^.^35^ The optimum conditions to maximise germling production for S. muticum were found to be a 15–30 min desiccation period in the shade, followed by immersion into normal salinity seawater 35°/_oo_ at 20 °C and illumination at 50–100 µmol photons m^−2^ s^−1^.^36^

Seaweed needs to be decontaminated prior to juvenile production for ‘pure culture’ and minimisation of predators and epiphytes (37), and is achieved by: (a) selection of fronds with a minimum of epibionts; (b) physical removal of epibionts; and (c) chemical disinfection.^38^ The most common chemical disinfectants utilised in macroalgal culture are sodium hypochlorite, reactive oxygen and organic solvents followed by an antibiotic wash, but care must be taken as seaweeds are highly susceptible to chemical damage due to the lack of a protective cuticle.^38^

An extensive description of how to establish a seaweed culture laboratory and the basic resources needed together with a culture system ‘roadmap’ for the production of young seed plants has recently been produced by the University of Connecticut.^39^

The successful cultivation of commercially important seaweeds such as Laminaria and Porphyra in Asia was only possible after life cycles were first understood and incorporated into the production of ‘seed’ in nursery operations.^19^ The summer sporeling method is based on the life history of the seaweed, and requires cultivation of mature sporophyte, collection of zoospores in early summer, cultivation of juvenile sporelings and transplantation of sporelings.^40^ Although the sexual propagation of Laminaria by this ‘summer sporeling method’ produces in China 800 million sporelings per year, it is a seasonal and time consuming process. The production of juvenile algae also represents a major cost component of the overall production; for example, this part of the process accounts for 30% of the cost of producing red seaweeds for carrageenan.^20^ Indeed, Kraan^7^ has concluded that juvenile sporophyte production is a major bottleneck in establishing macroalgae farming for biofuel production.

In sexual propagation a number of sporophytes are mixed to collect zoospores, which inevitably results in the mixing and degeneration of commercial seaweed species.^40^ Seaweed gametophyte cloning techniques have recently been developed (current examples: Gracilaria and Eucheuma), which may give greater species control, allow for crop improvement through breeding and selection, and permit sporophytes to be produced at any time of year.^39^, ^40^

### Growing‐on

Once the juvenile algae have been produced they must be ‘grown‐on’ just as with terrestrial plants. In this context, it is possible to envisage seaweed being cultivated in a number of ways:

- in land‐based tanks;
- intertidal;
- offshore deep‐sea;
- near‐shore.

The correct siting of cultured‐seaweed farms will be vital to ensure sufficient light (considerable reductions in yield with increasing depth are observed) and nutrients, while minimising disruption to other activities and the environment.^28^, ^35^, ^41^, ^42^ Locations with a flow‐rate of 5–10 cm s^−1^ have been suggested as being optimal for seaweed culture,^28^ while the minimum annual light requirement to support the growth of mature Laminaria on drop‐lines has been reported as being ∼ 70 mol photon m^−2^.^42^ Optimal conditions will vary with species. The University of the Highlands found that ‘sheltered waters’ that favoured the growth of both L. digitata and Saccharina latissimi were not suitable for Alaria which favoured more exposed sites (McEvoy, 2015, private communication).

As with terrestrial plants the timing of ‘planting out’ will have an influence on yield and economics. In Shetland seeded cultivation ropes deployed at sea in November produced greater yields and suffered less contamination from competitors and epiphytes than those deployed in February. However, the growth lines were exposed to the force of a Shetland winter (McEvoy, 2015, private communication).

#### Land‐based tank growth

Land‐based pond systems are currently used for the growth of macroalgae for the production of speciality seaweed products and have been considered for macroalgal cultivation for biofuel.^19^ Advantages of the land‐based systems over those based on water are:

- ease of plant management;
- ability to use seaweed without holdfast (the specialised ‘root‐like’ structure on the base of the seaweed which attaches it to a surface, but unlike roots they are not primary means for water and nutrient uptake);
- ease of nutrient application;
- avoidance of open sea problems such as bad weather, disease, and predation;
- cultivation operations easily located close to biofuel conversion operations.^19^

Despite these obvious benefits the construction and operation of ponds is expensive in both capital and operating cost, and will involve the loss of terrestrial sites that could be used for other purposes.

#### Intertidal growth

Humans have exploited intertidal natural stocks of seaweed for food and agriculture for thousands of years,^43^, ^44^ and species are commercially harvested from ‘wild‐stocks’, such as Ascophyllum nodosum and Fucus.^45^ However, the commercial intertidal cultivation of seaweed is currently limited primarily to Eucheuma and Gracilaria in low labour‐cost countries such as Philippines, Vietnam and Thailand.^20^, ^21^, ^43^ Carrageenan is cultured in shallow waters on off‐bottom systems where cultivation lines hang between stakes pegged to the sea‐floor.^20^ The yields of Gracilaria from sub‐tidal cultivation systems have been found to be typically 69% greater, than those of intertidal regions. In addition to potential problems of lower yield and harvesting cost, there are concerns over the environmental impact of intertidal cultivation, such as the displacement of other seaweed species, sea‐grasses and marine organisms in the intertidal area of production.^17^, ^20^, ^29^, ^46^

#### Offshore growth

The operation of offshore seaweed farms was initially tested by the Marine Biomass Program more than three decades ago in deep waters off the coast of Southern California. However, difficulties were encountered with the stability of both the necessary supporting structures and the attachment of the kelp to the surface of the growth, primarily as a consequence of the forces experienced in the dynamic open‐ocean environment.^19^, ^47^ In contrast, some success was achieved with offshore growth of Laminaria hyperborea in the North Sea using a novel ring structure culture support system which appeared more resistant to the forces of the open‐ocean, but was more expensive than the types of structures used in the Marine Biomass Program.^19^, ^48^

A significant issue surrounding seaweed cultivation in offshore deep sea environments are the high costs due to the engineering challenges of operating in the deep sea.^19^ Seaweed produced in the open ocean was estimated to cost $0.31 kg^−1^ wet weight in 1981 equivalent to more than $1 kg^−1^ today.^49^ Furthermore, considerable additional engineering research is needed to design structures that will allow the seaweeds to survive aggressive ocean conditions.^43^, ^47^ In contrast to near‐shore cultivation, expenditure and energy penalties (in addition to weather‐related issues) required to transport deep ocean seaweed to any processing plant will be significant. The economic feasibility of far offshore floating cultivation systems is considered doubtful with current technology.^50^

#### Near‐shore growth

Seaweed cultivation is currently primarily limited to near‐shore systems with installations being used for macroalgae culture for non‐fuel products in a number of countries particularly in Asia.^47^ Carrageenan is cultured not only on intertidal systems, but also near‐shore using long lines supported by buoys.^20^ Cultivation of a variety of different seaweed species is currently most commonly achieved using long‐line structures, not dissimilar to the ropes used for commercial cultivation of mussels. Such structures consist of an anchorage point, connected to a header rope on or near the surface of the water, which is supported by buoys, in turn this is connected to the main growing line.^28^ The capital costs of such a system which also facilitates mechanical harvesting were estimated to be $11 362 ha^−1^ in 1984^51^ (equivalent to ∼ $26 000 in 2016). Energy costs for near‐shore cultivation have been estimated at 2.15 MJ kg^−1^ dw.^52^ Other near‐shore growing methods include nets^53^ and rings^47^, ^48^ and 2 m × 10 m flat cultivation sheets.^54^ The cost of kelp grown commercially in China is ∼ £0.35 kg^−1^ dw.^55^ In China farming takes place near‐shore and relies heavily on manual labour, but cultivation in European waters, such as the North Sea will require a degree of mechanisation that has not yet been seen in China.^56^

Irrespective of the roping method, the ideal depth of the growth system has to be optimised for the particular seaweed species being grown, something that is further complicated by light availability and water clarity. Typically depths for long lines are 1–2 m for cultivation of kelp species with depth adjustment to optimise growth.^39^ Compared with the highly controlled tank culture methods growing seaweed on ropes is less labour intensive as once planted out the seaweed requires little attention, with only regular checks required to ensure that there is no loss or damage from storms, vandalism, or passing boats and occasional thinning of seaweed fronds.^39^

Offshore wind farms may offer ideal sites for macroalgal cultivation as the area is closed to shipping and the multi‐functional use could reduce capital and operational costs.^21^, ^49^ The siting of seaweed farms adjacent to existing aquaculture such as mussel or salmon farms, in what has been termed, Integrated Multi‐trophic Aquaculture (IMTA), could also have significant economic and environmental benefits.^23^, ^41^, ^57^, ^58^, ^59^ A 3 year break‐even price for seaweed cultured in IMTA associated with an existing mussel farm was estimated at ∼£1 kg^−1^ wet weight.^60^

### Yield from seaweed cultivation

The majority of productivity data for the cultivation of seaweeds is reported in terms of production per metre of culture line (kg m^−1^) rather than per unit area,^15^ and often without sufficient detail for calculation of areal productivity. A summary of areal productivity for a range of seaweeds is given in Table 4.

**Table 4:** Annual seaweed yields

	Wet weight yield	Dry weight yield	VS yield	
Species	kg m^−2^ yr^−1^	kg m^−2^ yr^−1^	kg m^−2^ yr^−1^	Ref.
Brown algae		3.3–11.3		70
Brown algae		3.3–11.3		71
Brown algae		3.3–13.1		12
Brown algae			2.6–8	30
Natural seaweed stands		3.6		49
Natural seaweed stands		0.1–4		8
Natural seaweed stands	12.5			15
‘Carrageenan seaweed’		0.6–10.8		20
Alaria esculenta		0.15–0.2		7
Gracilaria chilensis		14.5		55
Macrocystis pyrifera			3.7	72
Macrocystis pyrifera		3.8–6.2		7
Macrocystis pyrifera		6		55
Laminaria japonica			2.7	72
Laminaria japonica		6		8
Saccharina japonica		1.3–13.1		7
Saccharina japonica		2.2–3		15
Saccharina latissima		2		7
Saccharina latissima	4.6			62
Saccharina latissima	20			15
Saccharina latissima	6–14			63

Natural seabed annual areal seaweed growths of 12.5 kg wet algae m^−2^ yr^−1^ have been reported for L. hyperborean L. digitata and S. latissima equivalent to ∼20 tonnes ha^−1^ yr^−1^.^15^ However, yield can vary significantly, depending on nutrients and conditions, between 1 and 40 t dw ha^−1^ yr^−1^.^8^ Site and conditions will have a significant effect on naturally occurring and cultivated seaweed yields. Kerrison et al. 15 recently reviewed site selection for three species of seaweed in relation to the important physical and chemical parameters: temperature, salinity, water motion, nutrient concentrations, carbon dioxide, pH, light and ultra‐violet radiation.

Yields for farmed brown seaweed have been suggested to be in the range 26–80 t VS ha^−1^ yr^−1^,^30^ and 30.3–131 t dw ha^−1^ yr^−1^.^12^ In the beginning of the US marine biomass programme (1968–1990)^61^ yields of 140 t ash free dw ha^−1^ yr^−1^ were suggested, but this was considered unrealistic and yields of 23–34 t ash free dw ha^−1^ yr^−1^ were considered as more commercially viable.^51^ The productivity of S. japonica cultivated on lines has been reported as equivalent to 22–30 tonnes dw ha^−1^ yr^−1^,^15^ but under experimental conditions in China yields of up to 60 t dw ha^−1^ yr^−1^ have been achieved.^8^

In Europe yields of S. latissima cultivated on ropes equivalent 5.6–140 t fresh wt ha^−1^ yr^−1^ have been achieved,^62^, ^63^ and a yield 20.3 kg m^−2^ of wet material on a 5 m diameter offshore ring in the North Sea.^15^

A yield of 30 t dw ha^−1^ yr^−1^ (3 kg m^−2^ yr^−1^) would appear to be a reasonable but challenging target for UK seaweed cultivation. This is equivalent to an overall synthetic photosynthetic conversion of 1.3–1.9%, assuming a typical annual solar insolation in the UK of 700–1000 kWh yr^‐1^, 64 and a HHV of 16 MJ kg^−1^.^6^ In land plants grown for biofuels a photosynthetic efficiency of 1% is considered to be the best currently achieved in commercial production, but a doubling to 2% on a large scale could reasonably be aspired to in the future.^65^ Although yields based on a photosynthetic efficiency of 5% are possible for microalgae, the published data suggest that current practical photosynthetic efficiencies for the growth of microalgae are 2 to 3%.^66^, ^67^, ^68^, ^69^

## HARVESTING

An extensive study on the production of bioenergy (biogas and bioethanol) from brown seaweed concluded that such processes will only be economically viable if the costs of harvesting the biomass are low.^71^ Harvesting costs have been estimated to be up to 40% of the total cost of the production of biogas from seaweed.^51^

### Manual harvesting

Manual harvesting of seaweeds is common, and seaweed is currently mainly harvested by hand in the British isles,^21^, ^45^, ^73^, ^74^ but it is a labour intensive process.^19^ Indeed seaweed farming overall can be labour intensive; 36% of the total cost of carrageenan production in Indonesia is attributable to labour with harvesting labour cost being 22% of the total labour cost.^20^ In Mexico harvesting costs were 19% of the total cost of carrageenan production.^20^

### Mechanised harvesting

**Figure 2:**
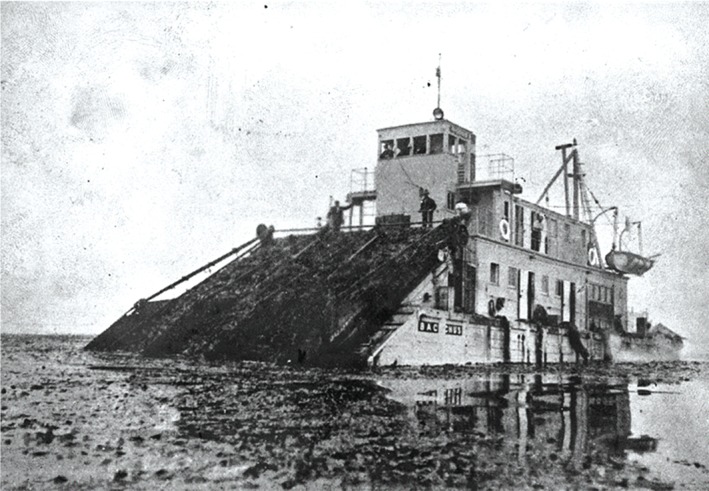
The Bacchus, one of three kelp harvesters designed by Hercules Powder Company engineers, c 1916, courtesy Steve Schoenherr.

To help to minimise harvest costs, a number of mechanised harvesting methods have been developed and explored, such as mowing with rotating blades, suction, or dredging with cutters, each of which invariably requires the use of boats or ships.^19^ Large mechanical harvesters for kelp were developed during the First World War (Fig. 2) with renewed development of the mechanisation of seaweed harvesting occurring in the mid‐1970s in France and Norway in response to the increasing demand for raw material for the alginate extracting industry.^75^ However, much of the mechanical harvest equipment appears to have been developed for wild harvest rather than harvesting line cultivated seaweed, and although boats, winches and cranes are used in the harvesting of cultivated seaweed, the systems currently used in Europe are still labour intensive.^56^, ^75^, ^76^ Although mechanical harvesting could reduce cost, manual harvesting could produce higher quality and more consistent feedstock as it may permit a greater degree of onsite removal of contaminates.^76^

Critchley *et al*.^77^ have reviewed the methods and cost of harvesting *S. muticum*, with the cost of harvesting by mechanical methods being estimated at £20–24 per tonne of wet algae collected (equivalent to £52–63 in 2015 based on Bank of England inflation data). The mass flow and energy required for the large‐scale mechanical harvesting of seaweed has been suggested as similar to that of large‐scale dredging, however no figures were quoted and it was concluded that much more research is needed.^78^ Energy cost of 5.5 MJ kg^−1^ have been suggested for harvesting macroalgae.^52^ Apart from the cost and energy efficiency issues, the main problem with these systems is the necessity for a boat, as seaweed harvesting is normally a seasonal activity, and therefore there is a need to find another use for the boat when not required for harvesting to maximise asset use and minimise cost.^45^

## POST‐HARVEST TREATMENTS

Once seaweed has been harvested it needs to be prepared for and transported to the upgrading facility where it is converted to the target fuel. There is considerable variability in the resistance to decomposition between seaweed types after harvesting. Some brown species are more resistant to decomposition than green seaweed, attributed to the presence of polyphenols, and can be stored at ambient temperature for hours or even days without starting to deteriorate.^45^ However, the seasonal nature of seaweed growth requires that methods of storage or preservation for longer periods must be developed to enable its use in year‐round fuels manufacturing and processes.

Once harvested, but before subsequent use, seaweed is first treated to remove foreign objects and debris by mechanical means or washing.^79^ Contaminants of the harvested fresh seaweeds may come from the farming environment (other varieties of seaweed (weeds), shells, sand, stones and mud) or from the farming system such as parts of growth system ropes.^20^ Although salt and other minerals may also be considered an impurity,^20^ their removal may not be necessary for some biofuel conversion processes, such as anaerobic digestion (AD), but their presence may impact directly on any chemical or thermochemical processing methodologies, such as liquefaction and gasification. If salts do need to be removed such operations will have a considerable effect on water usage, effluent production and overall process energy input requirements.

Commonly chopping or milling of the treated biomass is then required to increase the surface area to volume ratio that will improve the efficiency of combustion, AD and the hydrolysis of complex carbohydrates to sugar for fermentation.^79^, ^80^ Maceration prior to ensilage has been shown to increase fermentation rate and lactic acid concentration,^81^, ^82^ reduce ethanol production during ensilage,^83^ and reduce leachate losses during the ensiling of seaweed.^84^

### Transport of biomass

After harvesting the seaweed will need to be transported by boat or barge to the shore, and once it arrives on shore it will need to be transported to the ensilage, storage and gasification plants. The low energy density of biomass and its often dispersed geographically locations can cause transport costs to rise rapidly with size of biomass conversion facility.^85^

The fuel consumption of transporting biomass by barge at sea has been estimated to be 0.11 L diesel km^−1^ t^−1^ biomass^86^ and the fuel consumption of a 28.5 tonne truck transporting wood on public roads is 0.012 L biodiesel km^−1^ t^−1^.^87^ The HHV of biodiesel is 36.5 MJ L^−1^,^88^ therefore, the energy use to transport material by sea is 4 MJ t^−1^ km^−1^ and 0.4 MJ km^−1^ t^−1^ by truck on public road. A seaweed with a typical HHV of 16 MJ kg^−1^ dw^6^ and a moisture content of 82%^89^ would have an energy content of 2888 MJ tonne^−1^ wet, weight equivalent to the energy required for transport over 7000 km by road. However, the maximum economic distance to transport biomass for fuel is considered to be <50 km,^90^, ^91^ and Golberg *et al*.^92^ have suggested that the cost of transporting seaweed will provide a limit to the size of an algal biorefinery, and that optimal maximum collection distance is ∼40 km.

Taelman *et al*.,^27^ in a case study of the resources footprint of *Saccharina latissima* production near the west coast of Ireland, found the biggest potential to improve the footprint of seaweed production was reducing the fuel demand for transport (including harvesting and growth system maintenance), which contributed 44% of the total resource footprint.

### Preservation and storage

#### Drying and dewatering

Drying is a widely used ancient method of preserving food, feed and seaweed. Reducing the moisture content of the seaweed prevents both the growth of spoilage‐causing microorganisms and slows down detrimental enzymatic reactions.^20^ Sun‐drying is the main method of drying seaweed.^20^, ^52^, ^93^ Clearly this approach does not require fossil fuel energy, but is both weather and volume dependent. Sun‐drying in tropical locations may take 2–3 days in sunny weather, and could take up to 7 days in rainy seasons.^20^ Despite these limitations, solar methods are the least expensive drying option,^94^ but large areas are required as only around 100 g of dry matter can be produced from each square metre of sun‐drier surface.^95^

Finding a more controllable and cost‐effective method of large‐scale seaweed drying, compared with that of sun‐drying, is clearly key to establishing a viable seaweed‐to‐fuels processing industry.^20^ The removal of water from the algal biomass by evaporation is very energy intensive with the energy to heat water from 20 to 100 °C and evaporate it at atmospheric pressure requiring an energy input of ∼2.6 MJ kg^−1^.^11^ The water content of macroalgae (80–90%) is generally higher than that of many terrestrial crops (sugarcane ∼75%, grain maize 14–31%).^12^, ^31^, ^96^, ^97^ Thus, the energy to dry seaweed is higher than the Heating Value of dry seaweed.^45^, ^98^ Coal‐fired driers have been used in Ireland for the production of seaweed meal products to achieve a moisture content ∼10%, but this approach is uneconomic for biofuel production.^45^

Dewatering (the mechanical removal of water) generally uses less energy than evaporation to remove water, and it would appear preferable to minimise the water content of the harvested algae prior to drying. The dewatering of algal biomass using pressing or centrifugation of seaweed biomass to 20–30% will increase ‘shelf‐life’ and reduce transportation costs.^45^

#### Ensilage

An alternative preservation method is ensiling. It is routinely used for the storage of forage for animal feed. In ensilage lactic acid fermentation under anaerobic conditions converts water‐soluble carbohydrates into organic acids, mainly to lactic acid. As a result the pH decreases and the moist crop is preserved.^99^ Typically, ensiling conditions are achieved from spontaneous anaerobic lactic acid fermentation that is initiated by naturally‐present bacteria on the crop.^100^, ^101^ Dewatering and demineralisation are inherent features of ensiling^102^ which may be useful in facilitating gasification of seaweed.

There are several ensiling methods that are routinely employed to achieve the necessary conditions including trench, bunker, silos, clamp or heap silage, and bale silage, each with the primary aim of excluding air during the ensiling process and subsequent storage.^103^ Capital costs vary considerably and advantages and disadvantages are claimed for each method.^101^, ^104^ Bale silage accounts for ∼20% of total silage made in England and Wales.^104^

Despite its widespread use in terrestrial agriculture there has been little research on how to preserve seaweed biomass year round in order to satisfy continuous process demand.^105^, ^106^, ^107^ However, ‘an understanding of ensiling of seaweed is absolutely crucial for a substantial seaweed biofuel industry’.^107^ There was some research on the ensilage of seaweed in the 1950s,^105^ and more recently work on lactic acid fermentation of seaweed for novel‐food production.^106^ A patent application was made in 2013 for methods of ensiling algae and uses of ensiled algae.^108^ In addition to the work being carried out by the MBC group project work has recently been carried out in Ireland on the effect of ensiling seaweed in anaerobic methane production, supported by a grant from Marine Renewable Energy Ireland (MaREI) Centre and Gas Networks Ireland.^107^

The energy loss in the conversion of simple sugars to the necessary lactic acid, calculated from their higher heating value (HHV), is small with the HHV of lactic acid being ∼96% of glucose and xylose. The energy loss in the ensiling of maize and sugar‐beet was described as negligible.^109^ An FAO report has suggested that silage fermentation can preserve >90% of the harvested energy from the original biomass within the silage.^110^ A report by Saskatchewan Ministry of Agriculture^103^ has suggested that dry matter losses from some methods such as pit and bales storage can be considerably higher at >20%, but a recent study of grass, lucerne and maize ensilage found average dry matter losses of 9–12% and suggested that typical losses for farm‐scale bunker ensilage is 8%.^111^ Effluent production is influenced by a number of factors including species, moisture content, climate and ensiling conditions, and data from one feedstock may be of little value in predicting effluent production for other feedstocks^102^ thus data from terrestrial crops may not be a valid indicator for seaweed ensilage.

A recent study on the ensilage of five species of seaweed found large amounts of effluents were formed during ensiling of seaweeds with silage effluent being 10–28% of the original ensiled biomass with effluent production being lower in those species with a higher total solids (TS) content.^107^ Losses of volatile solid (VS) during ensilage were lower at 3–19% of the original biomass VS.^107^ A recent study at Durham University, as part of the MBC project, found seaweed dry matter losses of 22.5% for the red seaweed *Palmaria palmate,* and 22% for the brown seaweed, *Laminaria digitate*.^112^ Leachate losses were 150 mL kg^−1^ for *Palmaria palmate* and 60 mL kg^‐1^ for *Laminaria digitate,* with both seaweeds becoming drier following ensilage. A study of the ensilage of the brown seaweed *Sargassum muticum* found similar leachate volumes of 68 mL kg^−1^ for seaweed fronds ensiled whole, but much lower leachate volume of biomass ensiled chopped 27 mL kg^−1^ (1–2 mm typical size).^84^ However, dry matter losses were substantially lower at 2.7–8.7%. The *S. muticum* became drier following ensilage with reduced ash and particularly salt (NaCl) content. There was little change in the C%, H% and N% of the organic matter in the biomass as a result of ensilage in both studies. There was no statistical difference between HHV of the *S. muticum* biomass dry matter before and after ensilage.

During ensilage virtually all the organic sulphur was removed from the biomass.^84^ Under anaerobic conditions, organic compounds containing sulphur are broken down by bacteria, forming intermediate sulphur‐containing compounds that ultimately form hydrogen sulphide using low molecular weight organic volatile fatty acids as electron donors.^113^, ^114^ Sulphur reducing bacteria present in silage^114^, ^115^ are the potential cause of the loss of sulphur from the ensiled *S. muticum*. Some lactic acid bacteria have also been reported to metabolise sulphur‐containing amino acids,^116^ and thus may contribute to the breakdown of organic sulphur compounds. Low ash, salt and sulphur feedstocks are favoured for both gasification and AD and thus ensilage may yield downstream process benefits in biofuel production. The production of H_2_S during ensilage will also have operational health and safety implications. The total energy loss in the *S. muticum* biomass was 0.2–8%.^84^ Energy losses from seaweed ensilage are relatively small, and therefore it may be an energetically viable method of preserving seaweed for biofuel production.

For grass silage at a TS of 25% very little effluent is produced,^117^ and the UK's Ministry of Agriculture, Fisheries and Food (MAFF)^104^ has recommended that in order to minimise effluent production wilting to at least 25% dry matter before ensilage is required. Such wilting processes before ensilage also increase the concentration of sugars, which enhances the ease with which fermentation of the biomass occurs, while also reducing odours.^104^, ^118^ However, only rapid wilting (<24 h) offers benefits as slow wilting can lead to rotting and increased organic material losses.^118^ The requirement to rapidly wilt seaweed, prior to ensilage, could have considerable influence on operation, economics and energy balance of macroalgal ensilage for biofuel production.

Ensiling as well as being a method of preserving feedstock may also provide potential benefits in the downstream processes for biofuel production. There has in the past been a presumption that ensiling can increase the methane production in AD from some terrestrial crops.^109^, ^119^ This presumption that ensiling generally improves methane yields is not supported by results of many practical studies.^109^, ^119^ However, ensiling does not appear to have a negative effect on biogas production from AD.^109^, ^119^, ^120^ Results on the five species of seaweed show that ensiling seaweed for 90 days can increase methane yields by up to 28% and compensated for volatile solid losses during ensiling.^107^ However, recent results on the ensilage of *S. muticum* have shown that ensilage had no statistical effect on methane yields.^84^

## BIOFUELS PRODUCTION

One way in which extraction of energy from macroalgae can be categorised is according to whether an initial drying step is required or not. This leads to two distinct groups of processes:

1. energy extraction methods requiring dry macroalgae
  - direct combustion
  - pyrolysis
  - gasification (conventional)
  - trans‐esterification to biodiesel;
2. energy extraction methods for wet macroalgae
  - hydrothermal treatments
  - fermentation to bioethanol or biobutanol
  - anaerobic digestion.

A summary of the potential methods of energy extraction is given in Table 5. The methods of energy extraction from macroalgae have been recently reviewed as part of the MBC project.^121^ It was concluded that it is probably too early, at the current stage of biofuel development, to select definitively what method or combinations of methods for obtaining energy from macroalgae will be commercially exploited.

**Table 5:** Methods of energy extraction from macroalgal biomass

Method	Utilises entire organic biomass	Requires biomass drying after harvesting	Primary energy product
Direct combustion	Yes	Yes	Heat
Pyrolysis	Yes	Yes	Primarily liquid by fast pyrolysis
Gasification	Yes	Yes^b^ (conventional)	Primarily gas
Biodiesel production	No	Yes c	Liquid
Hydrothermal treatments	Yes	No	Primarily liquid
Bioethanol production	No a	No	Liquid
Biobutanol production	No a	No	Liquid
Anaerobic digestion	Yes	No	Gas

a Polysaccharides require hydrolysis to fermentable sugars. Some of the sugars produced from the breakdown of seaweed polysaccharides are not readily fermented.

b Supercritical water gasification (SCWG) an alternative gasification technology can convert high moisture biomass.

c No current commercial process for the wet trans‐esterification of wet macroalgal biomass.

Direct combustion is, historically and currently, the main method by which energy from dry biomass resources is realised, providing heat or steam for household and industrial uses or for the production of electricity.^122^ Macroalgal combustion does not appear to have been greatly explored.^123^, ^124^ However, the high energy required to dry seaweed, the relatively low thermal values and high ash and sulphur content, which can cause fouling and corrosion of boiler and unacceptable emissions, could preclude direct combustion as an economic method of exploiting seaweed.^121^, ^123^, ^124^

The higher lipid content of some microalgae compared with macroalgae has focused much of the published research work on the production of biodiesel from microalgal lipids via trans‐esterification.^11^, ^125^, ^126^ Macroalgal biomass typically has lower lipid content, 0.3–6% compared with microalgae which can have >70%. Oil levels of 20–50% are common for microalgae, but more typically reach only 10–30% when grown under nutrient replete conditions.^21^, ^78^, ^127^, ^128^ Macroalgae would, therefore, not appear to be a suitable feedstock for the production of biodiesel via trans‐esterification.

First generation bioethanol, such as that produced from corn in the USA and sugarcane ethanol in Brazil, is now widely produced and used, and currently is the liquid biofuel with the highest production volume (> 90 GL).^129^, ^130^ Bioethanol can be readily used in current supply chains, with 86% of cars sold in Brazil in 2008 capable of using ethanol or a mixture of ethanol and fossil fuel petroleum.^131^ Brown, green and red algae have all been fermented to ethanol, but brown algae are suggested as the principal feedstock for bioethanol production because they have high carbohydrate contents and can be readily mass‐cultivated.^132^ Although polysaccharides are the predominant component of macroalgae making up to 76% of the total dry weight, and typically ∼50%,^76^ the polysaccharide composition of brown seaweed is different from that of terrestrial plants, with the major polysaccharides of brown algae being laminarin, mannitol, alginate and fucoidan.^132^ These algal polysaccharides have been found to be difficult to ferment using conventional bioethanol technology and require considerable pre‐treatment for the production of bioethanol.^133^, ^134^ Horn *et al*.^151^ concluded that a commercial industrial seaweed bioethanol process will require higher ethanol yields to be viable. However, large seaweed ethanol production facilities have been proposed in both Denmark^47^ and Japan, ‘Ocean Sunrise Project’,^135^ but the economic and energy feasibility of these schemes is unknown, and as yet there appears to be no large‐scale production of ethanol from macroalgae.^47^

While seaweed cultivation for bioethanol is being explored in Asia, Europe and South America it is biobutanol from macroalgae that is attracting research interest and investment in the USA.^136^ Butanol has been explored as a transportation fuel for around 100 years, and has been suggested as a biofuel with the potential, not only to augment, but even replace ethanol as a gasoline additive due to its low vapour pressure and higher energy density.^137^ Although biobutanol has been produced on a pilot scale from algal sugars,^137^ it has been concluded that significant improvements in yield and process costs are still needed to make industrial‐scale butanol from the fermentation of seaweed economically feasible.^138^

Hydrothermal processing is a high pressure process where ‘wet’ biomass is converted into primarily a stable liquid hydrocarbon fuel (bio‐oil) in the presence of a catalyst.^122^, ^139^, ^140^ The ability of hydrothermal liquefaction to handle wet biomass makes it one of the most interesting methods of producing biofuel from algae^141^ and hydrothermal treatment of algae has attracted research interest.^140^, ^142^, ^143^, ^144^ Hydrothermal liquefaction of biomass with a moisture content above 90% is believed to have an unfavourable energy balance,^145^ and reviews of thermal treatments for biofuel production have concluded that commercial interest in liquefaction is low due to the more complex feed systems and higher costs compared with those for pyrolysis and gasification.^122^, ^139^, ^146^ The production of biofuel from seaweed via hydrothermal treatment, thus will require considerably more research to reduce process costs.

Both gasification and anaerobic digestion have been suggested as promising methods for exploiting bioenergy from biomass.^147^ A recent study that analysed four methods of microalgal bioenergy production found that anaerobic digestion produces more net energy than supercritical gasification, the latter requiring higher energy input and having a negative return on energy investment.^11^, ^148^ This conclusion is supported by a related study that has demonstrated that anaerobic digestion of ‘algal residues’, can have a higher net energy return and much lower greenhouse gas emissions than gasification.^149^ Despite the energy benefits from anaerobic digestion processes, gasification is a significantly more rapid process, which is a clear operational benefit, and if higher yields of combustible gas can be achieved through gasification then this may lead to a more favourable energy balance.

### Anaerobic digestion

Seaweed derived biogas was used industrially in the 19th century, and currently AD is perhaps closest to industrial exploitation.^121^, ^150^ Not only is it a relatively simple process from an engineering/infrastructure stance, but it has the potential to exploit the entire organic carbon content of macroalgae and can readily tolerate high moisture content without incurring additional process energy penalties. It is likely to play a leading role in combination with other methods, and could be the major method of biofuel production from macroalgae.

A report for the Crown Estates has concluded that AD at a small, distributed scale was economically feasible for the co‐digestion of seaweed with food waste.^150^ The gasification of seaweed with wood based biomass was also considered economically feasible. Conversely, the large‐scale anaerobic digestion or gasification of seaweed alone was considered extremely challenging economically, and will require seaweed delivered to the processing plant at below £300 per tonne.^86^, ^150^ Yokoyama *et al*.^72^ suggested that seaweed biogas will only be viable if the process also yields other high value products. However, the aim of the MBC is to produce liquid biofuel rather than gaseous fuels based on methane. Although, methane can be converted to the liquid fuel, methanol, the cost of production from biomass has been estimated at up to four times that from fossil fuel gas.^151^

### Gasification

**Figure 3:**
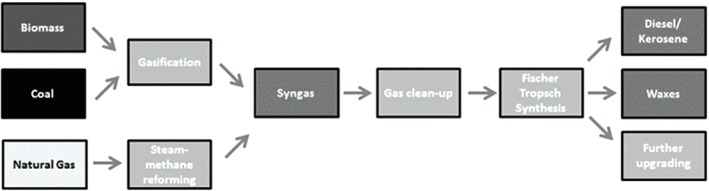
Stages involved in the overall FTS process from feedstock to products^121^

Gasification is the conversion of organic matter by partial oxidation at high temperature (800–1000 °C) mainly into a combustible gas mixture (syngas).^122^, ^139^, ^152^, ^153^ The syngas has a calorific value of 4–6 MJ m^−3^,^139^ and is a mixture of hydrogen (30–40%), carbon monoxide (20–30%) methane (10–15%), ethylene (1%), nitrogen, carbon dioxide and water vapour.^122^, ^152^ Syngas from gasification of biomass can be converted catalytically into hydrocarbons and water through Fischer–Tropsch synthesis (FTS),^154^ Fig. 3.

Gasification is generally a more rapid process than AD, but more energy input is needed to achieve the temperatures and pressure required compared with AD. Rowbotham *et al*.^155^ have suggested that thermochemical processing methods, such as gasification and hydrothermal liquefaction, are more applicable and versatile treatment options than AD and fermentation, due to the technological difficulties associated with treatment and refining to liquid fuels of complex, heterogeneous, multi‐component feedstocks, such as seaweed.

Conventional biomass gasification processes require dry feedstock.^156^ The gasification of dry lignite and woody biomass can have high yields with up to 90% of the original chemical energy in the biomass being recovered as energy in syngas,^157^ with the net energy return, including energy inputs, for pyrolysis operation of dry land agricultural biomass waste ranging from 42–53%.^158^ Studies on dried seaweed (*Laminaria digitata, Fucus serratus* and mixed macroalgae species from the Black sea) found that the syngas produced had low heating value compared with lignocellulosic materials due to a high proportion of carbon dioxide and carbon monoxide.^159^ However, seaweed is a high moisture feedstock and considerable energy needs to be used to dry it before conventional gasification, but supercritical water gasification (SCWG) is an alternative gasification technology for the conversion of high moisture biomass and it is suggested it can be net energy positive in a well‐engineered system.^160^ A methane‐rich gas has been obtained from *Ulva lactuca* by SCWG, and it is suggested that results indicate that the catalytic supercritical water gasification of macroalgae is feasible.^161^

It has been concluded that there is little data available on the gasification of algae, and in particular on the energy balance and the need for drying of algae prior to gasification.^94^ If gasification of macroalgae can be achieved using wet biomass it may be more economically and energetically attractive than traditional dry methods of gasification. The enthalpy change needed to take ambient temperature liquid water to a low‐density supercritical state (400 °C and 250 bar) is similar to that required to vaporise liquid water at atmospheric pressure but the advantage of the SCWG process is that much of the energy invested in reaching a supercritical state can be captured and used again, with the hot effluent from the gasification reactor being used to preheat the wet biomass feed stream.^156^ The reduced moisture content following ensilage^102^, ^112^ could be useful in reducing the energy required to dry seaweed prior to conventional gasification or the energy input required in SCWG. However, VS is also lost in ensiling in the leachate, and it may offset any energy gains unless it is utilised.

### Biorefinery

**Figure 4:**
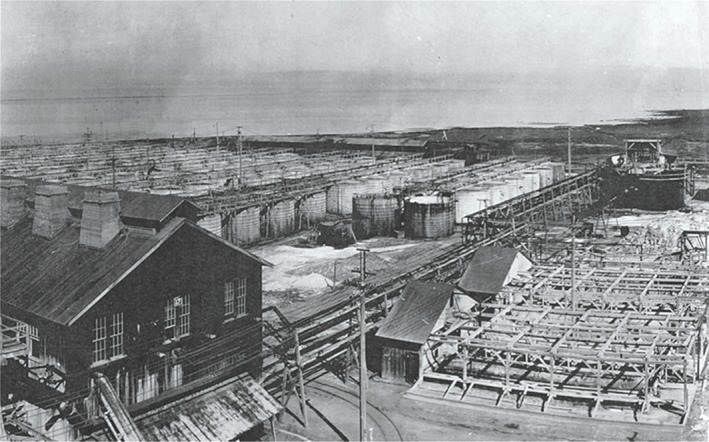
The Hercules kelp processing plant; courtesy of Steve Schoenherr.

A biorefinery concept that attempts to commercialise all the components of seaweed has been suggested as a more appropriate approach to the further exploitation of seaweed rather an approach solely for biofuel.^21^, ^59^, ^162^ One company, Hercules, was capable of producing 54 chemicals from seaweed during the First World War (Fig. 4), but closed shortly after the war when demand fell and alternative supplies became available.^8^ Seaweed has been used for the production of alkali, soda (sodium carbonate) and potash (potassium carbonate), for use in a variety of processes, but has again been replaced by cheaper sources of supply.^8^ However, seaweed has the potential to produce an additional wide range of high value biochemicals, nutraceuticals and pharmaceuticals^2^, ^163^, ^164^, ^165^ in addition to their current use for the commercial production of hydrocolloids.^166^

## PROCESS AND TECHNO‐ECONOMIC MODELS

There have been relatively few techno‐economic and LCA studies on macroalgae compared with microalgae.^121^ However, EnAlage has recently produced a freely available economic model for macroalgae production using Microsoft Excel.^167^ Although this model may be of value, the authors state; ‘Unfortunately, data from commercial seaweed farms are only available on a very limited scale’. There is also uncertainty concerning the scale‐up of the processes,^56^, ^167^ and thus there is a need for more data particularly at a commercial scale.

An LCA study, as part of the EnAlage project, concluded that seaweed can be cultivated with a comparable life cycle resource demand to several land plants, but energy return was marginal at best with an energy return on investment (EROI), the ratio of the energy produced compared with the amount of energy invested in its production, of 0.25–1.1.^27^ The EROI is useful measure of the viability of fuels: a ratio of less than one indicates that more energy is used than is produced, and an EROI of 3 has been suggested as the minimum that is viable.^168^ Energy return on energy investment (EROI) of seaweed ethanol has been estimated as being comparable with corn ethanol at 1.78,^169^ though more recent studies has suggested that algal bioethanol production will have an EROI <1.^16^, ^55^ Processes that use the entire biomass rather than just the fermentable sugars have more favourable EROIs^16^ with seaweed biogas having an EROI of 2.4 and a combined production of biogas and bioethanol from seaweed having an EROI of 3.0.^55^ However, these more favourable EROIs were achieved using labour intensive sub‐tidal shallow waters off‐bottom systems, and the EROI for offshore long line seaweed culture combined biogas and bioethanol production are lower at <1–2.^55^ Although LCAs are an accepted methodology for assessing the potential of algal energy production systems,^170^ Pfromm *et al*.^171^ have suggested that algal biofuel LCAs tend to focus on materials rather than processes, and the expansion of LCAs to provide energy balances may not be an optimal approach.^171^ In addition to the need for more data on seaweed biofuel there is also a need to develop alternative EROI models to those based on LCAs.

## CONCLUSION

The production of biofuel from seaweed is economically, energetically and technically challenging at scale. Processes that exploit the entire algal biomass such as AD, gasification or a biorefinery concept appear to offer the best chances of success. Ensiling appears to be a simple and relatively low energy means of storing seaweed which will be essential in providing continuity of supply to a continuous biofuel production process. There is a need for more quantitative data on all parts of the seaweed biofuel process especially at scale and for seaweed harvesting in particular. The additional data generated by the MBC project will allow the development of an energy balance model to consider a number of production process operations and identification of the most critical parameters affecting net energy production in growth, harvesting and seaweed energy utilisation; together with a techno‐economic analysis of macro algal biomass production. The success of any seaweed biofuel energy balance or techno‐economic assessment will depend on quality and quantity of the data produced.
